# EDNC: Ensemble Deep Neural Network for COVID-19 Recognition

**DOI:** 10.3390/tomography8020071

**Published:** 2022-03-21

**Authors:** Lin Yang, Shui-Hua Wang, Yu-Dong Zhang

**Affiliations:** School of Computing and Mathematical Sciences, The University of Leicester, University Road, Leicester LE1 7RH, UK; ly109@student.le.ac.uk

**Keywords:** COVID-19, CT scans, deep learning, transfer learning, ensemble, automatic recognition

## Abstract

The automatic recognition of COVID-19 diseases is critical in the present pandemic since it relieves healthcare staff of the burden of screening for infection with COVID-19. Previous studies have proven that deep learning algorithms can be utilized to aid in the diagnosis of patients with potential COVID-19 infection. However, the accuracy of current COVID-19 recognition models is relatively low. Motivated by this fact, we propose three deep learning architectures, F-EDNC, FC-EDNC, and O-EDNC, to quickly and accurately detect COVID-19 infections from chest computed tomography (CT) images. Sixteen deep learning neural networks have been modified and trained to recognize COVID-19 patients using transfer learning and 2458 CT chest images. The proposed EDNC has then been developed using three of sixteen modified pre-trained models to improve the performance of COVID-19 recognition. The results suggested that the F-EDNC method significantly enhanced the recognition of COVID-19 infections with 97.75% accuracy, followed by FC-EDNC and O-EDNC (97.55% and 96.12%, respectively), which is superior to most of the current COVID-19 recognition models. Furthermore, a localhost web application has been built that enables users to easily upload their chest CT scans and obtain their COVID-19 results automatically. This accurate, fast, and automatic COVID-19 recognition system will relieve the stress of medical professionals for screening COVID-19 infections.

## 1. Introduction

According to the most recent World Health Organization (WHO) data on 9 December 2021, the cumulative number of confirmed cases of COVID-19 disease globally reached 267,184,623. The number of deaths reached 5,277,327 cases [1].

WHO chief scientist Soumya Swaminathan suggested humans were about 60 percent of the way to fight the Coronavirus [2]. However, unexpected obstacles can still arise, such as the sudden emergence of new variants [3]. Since the outbreak of the Coronavirus pandemic, many variants of the virus have emerged. Compared to the original virus, the Delta variant has a 108% higher chance of being admitted to the hospital, a 235% increased risk of intensive care units (ICU) admission, and a 133% increased risk of death [4]. However, partial and complete vaccination can reduce the risk of severe illness and death for all variants of concerns [5]. The number of hospitalizations, ICU admissions, and deaths decreased throughout the study as vaccinations increased [4]. However, Swaminathan stressed that some regions of the world have very high vaccination rates of 70 to 80 percent [2].

In contrast, less than 4 percent of the population is vaccinated in other regions, such as Africa [6]. The more this situation is tolerated, the more likely new variants will emerge. Swaminathan called on certain countries not to promote vaccines among those who have already been vaccinated but to focus on immunizing the unvaccinated and ensuring that everyone has equitable access to the vaccine [2,7].

It is paramount for those areas that do not have access to vaccination to diagnose coronavirus patients quickly. Both quantitative reverse transcription-polymerase chain reaction (RT-qPCR) testing and CT imaging diagnosis play an essential role in screening COVID-19, with the latter serving as an important complement to the former [8,9,10]. The extent of the lesion detected on CT scans of the patient’s lungs is closely related to the severity of the COVID-19 disease [11]. CT is a method of scanning a particular part of the body one section after another, employing collimated X-ray beams, gamma rays, ultrasound, other radiation, and a pretty sensitive detector [12]. The data can then be combined with a computer to create cross-sectional images of the body, which can be further reconstructed into detailed 3D images or slices of different thicknesses as required [13]. Doctors may need a lot of time and effort to diagnose lung conditions from CT images. Fortunately, deep learning technology can help quickly diagnose the COVID-19 condition with CT scans.

Deep learning is currently a popular study tool for medical image analysis. It has the potential to minimize doctors’ diagnostic workload while also improving the speed at which they make decisions [14]. Deep learning may replace doctors’ long-term experience and thorough review in diagnosing a patient’s disease. Patients can rapidly obtain a more objective opinion after an assessment [15]. Under this pandemic, deep learning and CT scan images have allowed staff to promptly diagnose patients with suspected COVID-19 infection [16].

Various deep learning models have been built and successfully used to recognize COVID-19. A ResNet 50 model for identifying COVID-19 using chest CT scans was presented in Ref [17]. They provide the ResNet base model with the wavelet coefficients of the complete image without cutting any areas of the image. The accuracy of the result was 92.2%. Researchers in Ref [18] evaluated five deep CNN learning models, AlexNet, VGG16, VGG19, GoogleNet, and ResNet50, in detecting COVID-19 patients. The researchers utilized traditional image augmentation with CGAN to boost classification performance for all five models. The outcome suggested ResNet-50 has attained the highest accuracy of 82.91%. Eight pre-trained models including VGG16, VGG19, InceptionV3, InceptionResNetV2, Xexception, DenseNet121, DenseNet169, and DenseNet201 were examined for recognizing COVID-19 patients in Ref [19]. The results indicate that DenseNet201 achieved the highest accuracy of 85%. In Ref [20], a CNN design was proposed based on SqueezeNet for the rapid identification of COVID-19 CT images with regard to other pneumonia and healthy CT images. The architecture permitted an accuracy of 85.03%. Researchers in Ref [21] created FCONet, employing VGG16, ResNet-50, Inception-V3, or Xception as a backbone and using a dataset of 3993 CT scans for recognizing COVID-19. The result indicates that FCONet had an average accuracy of 96.97%. Ref [22] developed a simple CNN and modified pre-trained AlexNet model with a CT dataset of 361 CT images. The outcome suggested that modified CNN had attained the best accuracy of 94.1%.

The contributions of our paper are summarized as follows:

- We propose EDNC (F-EDNC, FC-EDNC, and O-EDNC) ensemble deep neural network for COVID-19 recognition, which helps clinicians rapidly and accurately analyze and recognize COVID-19 lung infections from chest CT scans.
- A deep neural network named CANet has been developed and built from scratch for comparative analysis with EDNC.
- Our proposed F-EDNC has achieved an accuracy of 97.55%, followed by FC-EDNC (97.14%) and O-EDNC (96.32%).
- A web application allows users to use F-EDNC easily.

The rest of this paper is structured as follows: Section 2 discusses materials and methods. Section 3 presents the results. Section 4 compares the results with state-of-the-art approaches. Section 5 concludes this study. 

## 2. Materials and Methods

**Figure 1:**
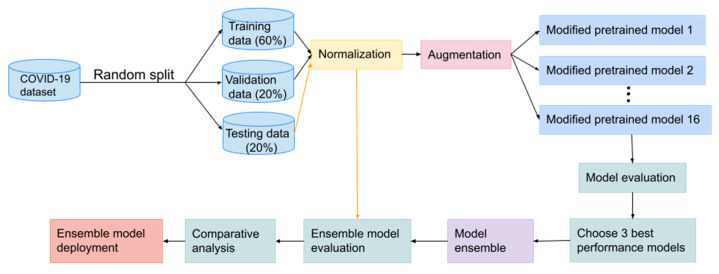
Flowchart of COVID-19 recognition system design.

This section focuses on the methodology of developing and implementing the COVID-19 recognition model. We present deep learning methods to distinguish between chest CT scans for COVID-19 and non-COVID-19 symptoms. The flow diagram shown in Figure 1 illustrates these key phrases.

### 2.1. The Dataset

#### 2.1.1. Main Dataset

The COVID-19 recognition task in this paper uses a CT scan dataset titled SARS-CoV-2, which is available at [23]. It contains 2481 CT scan images of both sexes collected from hospitals in Sao Paulo, Brazil. Of these CT scans, 1252 were COVID-19 positive, and 1229 were COVID-19 negative (not normal).

**Figure 2:**
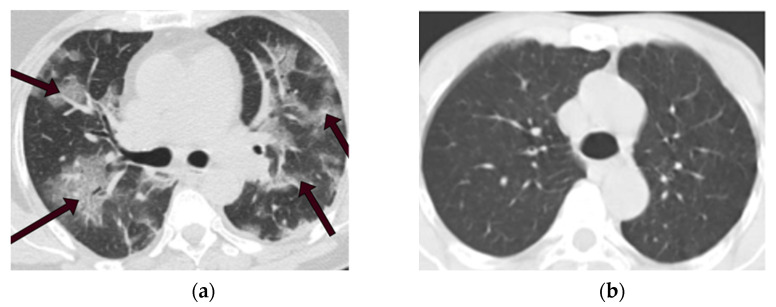
CT scans of COVID-19 and non-COVID-19 patients. (**a**) CT scan of a COVID-19 patient. (**b**) CT scan of a non-COVID-19 (not normal) patient.

These CT scan images are in PNG format with 104 × 119 to 416 × 512 spatial resolution. We selected 1229 images from each category to make the data balanced perfectly. Figure 2a displays a CT scan of a COVID-19 patient in this dataset, the area indicated by the arrows is infected with COVID-19. In contrast, a CT scan of a non-COVID-19 patient is depicted in Figure 2b. Table 1 indicates information on CT images used in this study.

#### 2.1.2. Alternative Dataset

**Figure 3:**
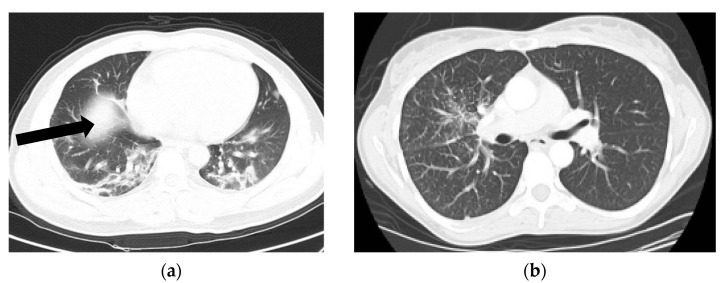
CT scans of COVID-19 and non-COVID-19 patients. (**a**) CT scan of a COVID-19 patient. (**b**) CT scan of a non-COVID-19 (not normal) patient.

In order to prove the generalization of the proposed deep learning models, another public CT dataset named COVIDx CT-2A [24] has been applied in this paper as shown in Figure 3. CT images numbering with PNG format have been randomly selected from COVID-19 and non-COVID-19 data. Table 2 indicates information on CT images used in this study.

#### 2.1.3. DICOM Format Dataset

The chest CT scan technology captures a series of sequential images from the patient’s lung. The infected spots may present in some images but not in others in an image series; for example, the lung is closed at the start and end of each CT scan image series. In order to detect COVID-19 symptoms effectively, a sample of data that indicated that the interior of the lung was clearly apparent in them is needed. Thus, to choose an image from each patient’s chest sequence images for training and validation purposes, only images in the middle of the CT sequence can be selected. Some previous methods of automatically selecting images inside the lung that are visible from a CT sequence have been used in [25].

If a user utilizes a CT dataset with DICOM format, a Python program of converting DICOM format to PNG can be executed as follows to feed the deep learning model:

Step 1: Read the DICOM image with the *dicom.read_file()* function.

Step 2: Translate the rescale slope and intercept information from the DICOM image header.

Step 3: Display the image in the proper range by using window (1500) level (−600) and width information from the image header.

Step 4: Convert the DICOM image to PNG format using the *cv2.convertScaleAbs()* function.

### 2.2. Data Preprocessing

First of all, the dataset (the selected chest CT scan dataset) is randomly split into training, validation, and testing sets with 60%, 20%, and 20%, respectively. Secondly, to train the deep learning model appropriately, we rescale the pixel values of images to the range of [0, 1] from [0, 255] due to the pixel-value representation required in image processing [26], which can be described as follows:

(1) $$I_{scaled}=\left(I-Min\right)\frac{Max_{new}-Min_{new}}{Max-Min}+Min_{new},$$

where Min and Max represent pixel values of 0 and 255, and $Min_{new}$ and $Max_{new}$ are the new pixel values of 0 and 1. This pixel-value rescaling approach is conducted in all training, validation, and testing datasets.

Further, the deep learning network requires fixed-sized data; thus, the sizes of all CT images are rescaled to 224 × 224 to meet the input size requirement [27]. Moreover, a larger dataset in deep learning may yield higher classification accuracy than a smaller dataset. However, having a large dataset is not always practical [28,29,30,31]. Thus, a data augmentation approach is used to increase the volume of data without acquiring new images [32]. To augment CT scan images used in this work, we perform geometric alterations such as picture rotation and flipping.

### 2.3. Modelling

#### 2.3.1. Transfer Learning Models

Given the shortage of CT scans of COVID-19 patients, training a Convolutional Neural Network (CNN) from scratch may be challenging. To overcome this difficulty, we use transfer learning techniques and a range of pre-trained models [33]. The primary advantage of transfer learning is that it can train data with fewer samples and less time [34]. The knowledge learned from the previously trained model can be transferred to the newly trained model [35].

Sixteen widely used CNN models are chosen to perform transfer learning for the COVID-19 recognition task: VGG16, InceptionV3, ResNet50, ResNet152V2, ResNet101, ResNet101V2, DenseNet201, MobileNetV3 small, MobileNet, MobileNetV2, VGG19, ResNet50V2, XceptionNet, InceptionResNetV2, NASNet, and EfficientNet. The reason for selecting these models is that they are effective in computer vision. Many of them have been reported to function well in medical diagnostics [36,37].

**Figure 4:**
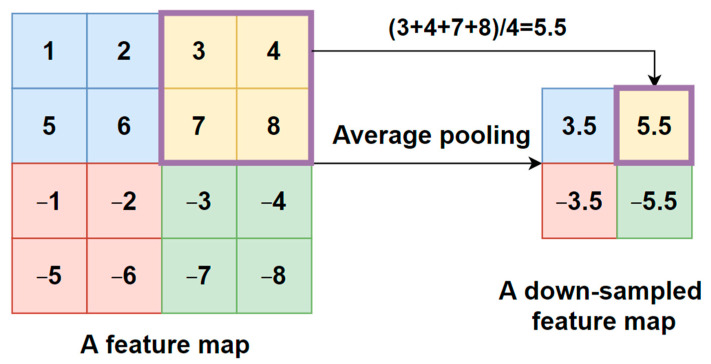
Average pooling procedure.

These models have been pre-trained on the ImageNet dataset for classification purposes. ImageNet is a freely accessible image database that contains 14 million photos classified into 20,000 categories [38]. Due to the enormous dataset utilized to train these sixteen models, the learning weights of these models may be used to recognize pictures in the medical sector. The above sixteen models are used as the base models. Thus, the feature extraction layers (convolutional and pooling layer pairs) are frozen to keep their ImageNet-optimized weights, avoiding information loss and maximizing feature extraction capabilities for future COVID-19 tasks training. Then, the initial fully connected layers of the pre-trained models are trimmed, and the following layers are added to classify COVID-19 CT scans:

- An average pooling layer, which produces a down-sampled feature map by averaging the values of all pixels in each batch of the feature map, and the calculating procedure is shown in Figure 4. The output size of the pooling layer is calculated as follows:
  (2) $$O_{p}=\lfloor \frac{W_{I}-F}{S}+1\rfloor ,$$
  where $W_{I}$ represents the input size of the pooling operation. F is the size of the pooling filter. S is the stride size;
- A flattened layer to convert the down-sampled feature map to a one-dimensional array;
- A fully connected layer with 64 filters and a Rectified Linear Unit (ReLU) activation to connect each neuron in layers before and after. ReLU helps to solve the problem of vanishing gradients. It is calculated using the equation below.
  (3) $$f\left(x\right)=\max\left(0,x\right).$$
- A dropout layer with a 0.5 dropout ratio to mitigate model overfitting problems;
- An output layer with Softmax activation to identify whether a CT-Scan is positive or negative for COVID-19 diagnosis [39,40,41,42]. Unlike ReLU, Softmax is frequently used for classification in the last layer of a model. It can be written as the following equation.
  (4) $$f_{i}\left(\vec{a}\right)=\frac{e^{a_{i}}}{\sum_{k}^{K}e^{a_{k}}},\;\;i=1,2,\ldots k.$$

**Figure 5:**
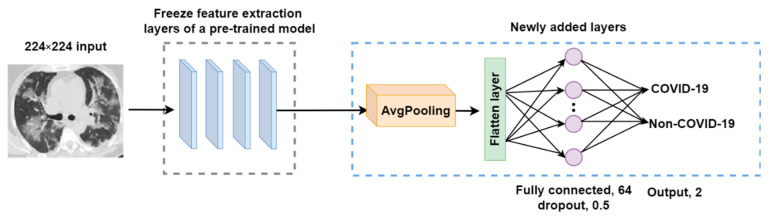
The architecture of the modified pre-trained model.

These newly added layers within the modified models are trained using the COVID-19 dataset. After each training epoch, the models are validated against a validation set. Since this research examines a binary classification problem (COVID-19 and non-COVID-19 classes), the number of neurons at the output layer was, thus, set to two. Figure 5 shows the modified architecture of the sixteen pre-trained models used in this research.

#### 2.3.2. The Proposed EDNC Architectures

The accuracy of disease prediction is critical in the medical field since erroneous choices result in high expenses and risks to human life. The drawback of using the predictions of several deep learning classifiers independently is that they have a significant degree of variation. Because each model is designed differently and trained independently, they update their weights separately and provide inconsistent results when asked to categorize the same data. These problems may be addressed by the ensemble of individual models, which will decrease variance, and the ensemble model will be more generalizable than the individual models [43].

The ensemble deep neural network for COVID-19 recognition (EDNC) models are proposed based on the combination of three pre-trained models. Three out of sixteen best-performing pre-trained models are chosen to execute the ensemble. Similarly to the individual transfer learning model, feature extraction layers in each of the three pre-trained models were set to be untrainable, preventing the weights from being changed in new model training. The next section details the architectures of three types of combined models.

##### F-EDNC

The primary goal of the feature-ensemble deep neural network for COVID-19 recognition (F-EDNC) is to group more characteristics from the input to the model. Thus, this proposed feature-ensemble technique produces a dataset that combines all desired features in the same CT scan input. It comprises three pre-trained models with the highest accuracy for identifying COVID-19 images. Assume $I_{input}=\left\{i_{1},i_{2},\;\ldots ,i_{n}\right\}$ to be the input COVID-19 image dataset, then we have the following:

(5) $$W_{F}=\left\{w_{F1},w_{F2},\ldots w_{Fn}\right\},$$

where $w_{F1},w_{F2},\ldots w_{Fn}$ are the feature extracted by the transfer learning models from the same input $i_{n}$. In this case, n is 3.

Therefore, the feature ensemble from different pre-trained models can be represented as follows.

(6) $$E_{F}=Concatenate\left[w_{F1},w_{F2},\ldots w_{Fn}\right].$$

These three models were trimmed after feature extraction layers with average pooling layers added. Following that, an ensemble layer was created to merge the outputs of these three feature extraction layers to obtain more precise data with respect to feature information. Then, three layers were added to complete the feature ensemble model: a flatten layer, a fully connected layer, and an output layer. The loss function we used for our model is categorical cross-entropy, which can be represented as the following equation:

(7) $$L_{i}=-\underset{j}{\sum }y_{i,j}\log\left({\hat{y}}_{i,j}\right),$$

where j represents the label, y are the target values, and $\hat{y}$ are the predicted values.

**Figure 6:**
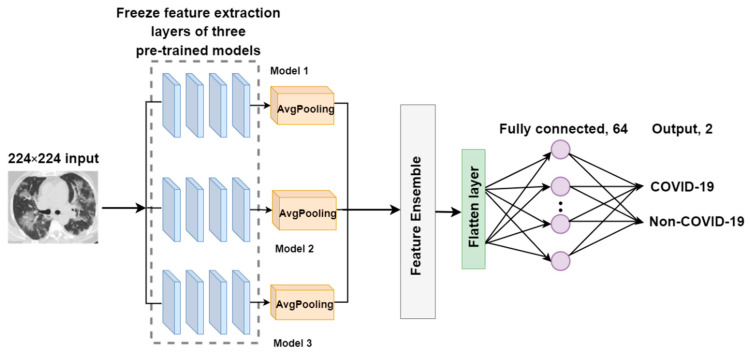
The architecture of the F-EDNC model.

The architecture of the F-EDNC model is illustrated in Figure 6.

##### FC-EDNC

The fully connected-ensemble deep neural network for COVID-19 (FC-EDNC) combines the fully connected layers of three pre-trained models to create an ensemble model with 386 trainable parameters. The output of fully connected layer $w_{FCn}$ of the pre-trained models will be utilized as a distinct input for this proposed model. In this case, n is 3.

(8) $$W_{FC}=\left\{w_{FC1},w_{FC2},\ldots w_{FCn}\right\}.$$

Therefore, the fully connected layer ensemble from different pre-trained models can be represented as follows.

(9) $$E_{Fc}=Concatenate\left[w_{Fc1},w_{Fc2},\ldots w_{Fcn}\right].$$

**Figure 7:**
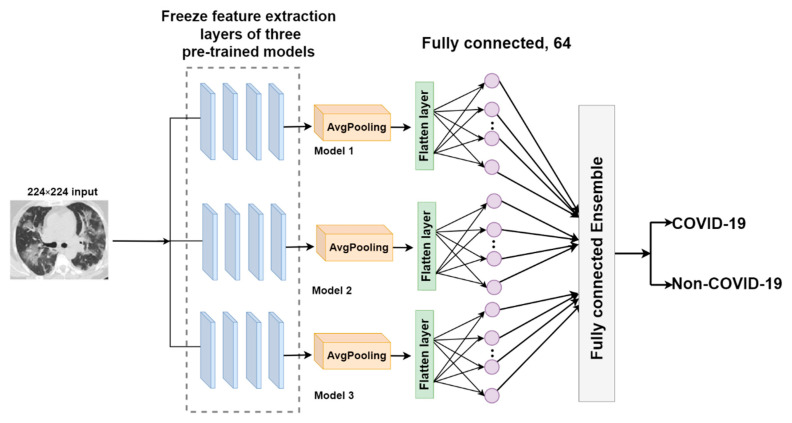
The architecture of the FC-EDNC model.

The output of fully connected layers is concatenated to generate a more accurate probability of identifying COVID-19 CT scans. The architecture of the FC-EDNC model is indicated in Figure 7.

##### O-EDNC

The output-ensemble deep neural network for COVID-19 (O-EDNC) approach is accomplished by assemble three pre-trained models at the output layer with 14 trainable parameters. This method can be represented as follows:

(10) $$W_{O}=\left\{w_{O1},w_{O2},\ldots w_{On}\right\},$$

where $w_{O1},w_{O2},\ldots w_{On}$ are the outputs of individual pre-trained models.

Thus, the output layer ensemble from different pre-trained models can be represented as follows.

(11) $$E_{O}=Concatenate\left[w_{O1},w_{O2},\ldots w_{On}\right].$$

**Figure 8:**
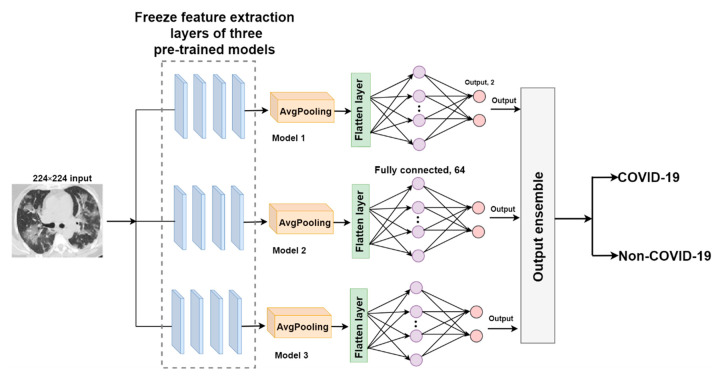
The architecture of the O-EDNC model.

This method assumes that the ensembled model may learn more characteristics in this merged output to make more precise predictions. The architecture of the O-EDNC model is shown in Figure 8.

#### 2.3.3. CANet: A Self-Build CNN Model for Comparative Analysis

**Figure 9:**
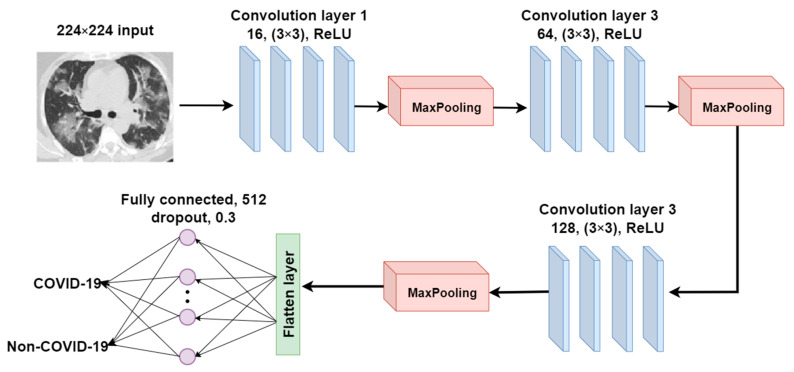
The architecture of the CANet model.

We proposed a CNN model and built it from scratch to compare it with the pre-trained and ensemble models in terms of model performance, training time, and model complexity in the COVID-19 recognition task. This proposed CNN model, CANet, is constructed using three convolutional and max-pooling layers, as shown in Figure 8. The 2D convolutional operation can be written as follows:

(12) $$s\left[i,j\right]=\left(I*K\right)\left(i,j\right)=\underset{m}{\sum }\underset{n}{\sum }I\left(m,n\right)K\left(i-m,j-n\right),$$

where I represents the input image, K is the kernel, and * represents the convolution operation. The number of filters in the first convolutional layer is set to 16 and raised to 64 and 128 in subsequent convolutional layers. The filter’s size is set to 3 ∗ 3. Each activation unit in the convolution layers has been implemented using ReLU activation. The output size of the convolutional layer is calculated using the following equation:

(13) $$O_{c}=\lfloor \frac{W_{I}-F+2P}{S}+1\rfloor ,$$

where $W_{I}$ represents the input size of the convolution operation. F is the size of the convolution filter. S is the stride size, and P is the padding size. The architecture of the CANet model is shown in Figure 9.

Three layers (a flatten layer, a fully connected layer, and a dropout layer) and a Softmax activation function are added to complete this CNN architecture. The model is trained and assessed in 50 epochs using the same COVID-19 dataset as the transfer learning models.

### 2.4. Localhost Web Application Development

#### 2.4.1. Web Application Workflow

**Figure 10:**
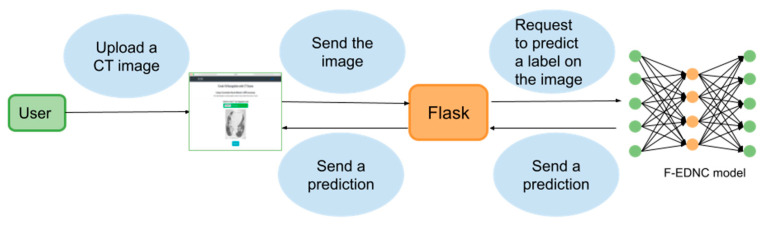
The architecture of the COVID-19 recognition system.

A web application is built based on the Flask framework, enabling users to upload their CT images easily and to obtain their COVID-19 results quickly. The architecture of the frontend and backend of COVID-19 recognition system is shown in Figure 10.

The following steps detail the functionality of the web application.

Step 1. The user visits the web application and uploads a CT image.

Step 2. The submitted picture is sent to the backend to which the proposed F-EDNC model is supplied. The image is resized to 224*224 and is converted to a NumPy array containing the pixel intensities before feeding into the model.

Step 3. The F-EDNC model has been saved in HDF5 format in the backend and is loaded by the *model.load()* function to process the input image.

Step 4. The output is calculated by the *predict()* function with the NumPy size array (2,1), which contains the two classes of probability. The highest probability class is then retrieved.

Step 5. The result is displayed at the front end.

#### 2.4.2. Technology Used in Building the Localhost Web Application

Technologies such as Python, Keras, Tensorflow, NumPy, and Pandas are used in building the backend model. The Flask framework routes the web page and hosts the web server in Python. The advantage of using Flask is that it can build a web application in one single python file; moreover, it reduces the work of coding in JavaScript and jQuery. To develop a web application that recognizes CT scan images, we create two routes on the flask application: an index page route for the users to upload their image file and a predicted route to predict the saved model. Furthermore, Bootstrap was utilized as a CSS stylesheet in building a webpage. Bootstrap is a CSS framework that provides some pre-built CSS classes. It can help incorporate responsive web pages in the web application so that our web pages can work well on mobile browsers.

### 2.5. Model Evaluation

#### 2.5.1. Experimental Setup

The networks are implemented on Jupiter notebook with Python 3.7 (Python Software Foundation, Leicester, UK), TensorFlow 2.4.0 (Google, Leicester, UK), Keras 2.3.1 (François Chollet, Leicester, UK), and Scikit-Learn 0.20.4 (David Cournapeau, Leicester, UK). They are trained on a PC with Intel CPU Xeon E5–2680 v2, 16 GB RAM (Intel, Leicester, UK), and Nvidia GPU RTX2070S (Nvidia, Leicester, UK). The web application is developed with Flask 1.0.2 (Pallets, Leicester, UK).

#### 2.5.2. Confusion Matrix

**Figure 11:**
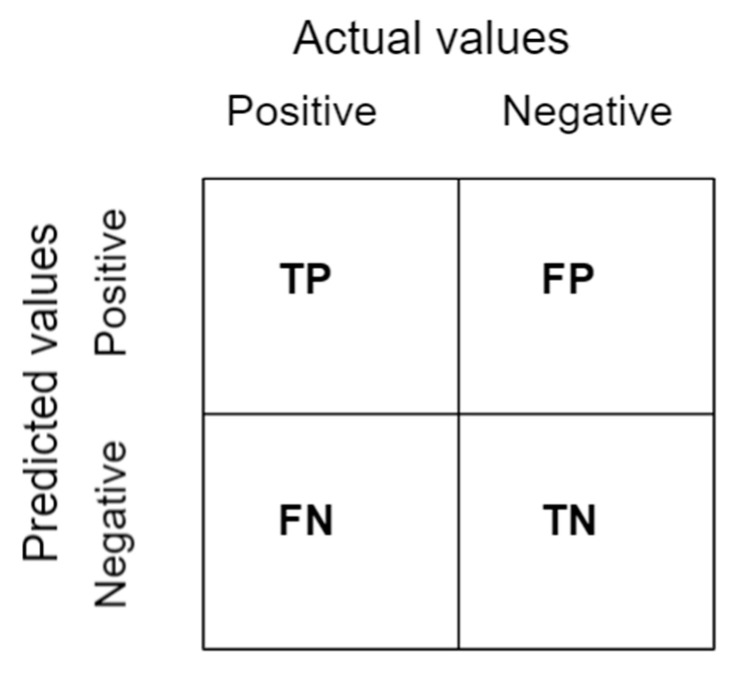
A representation of the confusion matrix.

A confusion matrix is a table as shown in Figure 11 that summarizes the results of a classification problem prediction [44]. The number of right and wrong predictions is combined and classified in four distinct ways as follows:

True Positive (TP): The prediction and the actual outputs are both positive.

False Positive (FP): The prediction is positive, but the actual output is negative.

True Negative (TN): Both the prediction and the real result are negative.

False Negative (FN): There is a negative prediction, while the actual result is positive. 

#### 2.5.3. Classification Metrics

The following five metrics were used to evaluate the model’s performance [44]:

Accuracy refers to the ratio of correct to incorrect predictions.

(14) $$\mathrm{Accuracy}=\frac{\mathrm{TP}+\mathrm{TN}}{\mathrm{TP}+\mathrm{FP}+\mathrm{TN}+\mathrm{FN}}.$$

Precision indicates the accuracy of which a model classifies a sample as positive.

(15) $$\mathrm{Precision}=\frac{\mathrm{TP}}{\mathrm{TP}+\mathrm{FP}}.$$

Sensitivity refers to a model’s ability to recognize positive samples.

(16) $$\mathrm{Sensitivity}=\frac{\mathrm{TP}}{\mathrm{TP}+\mathrm{FN}}.\;$$

F1-Scores measures precision and recall in a balanced manner.

(17) $$F1=\frac{2\times \mathrm{Precision}\times \mathrm{Recall}}{\mathrm{Precision}+\mathrm{Recall}}.$$

Specificity counts the number of negative samples that have been identified as such.

(18) $$\mathrm{Specificity}=\frac{\mathrm{TN}}{\mathrm{TN}+\mathrm{FP}}.$$

## 3. Results

### 3.1. Results of Sixteen Modified Pre-Trained Models

The main dataset is randomly partitioned into training, validation, and test subsets with 60%, 20%, and 20%, respectively. The proposed models are trained using training data and validated against the validation set after each training cycle. Then, we use the testing dataset to evaluate the models and quantify the performance of the models using evaluation metrics.

#### 3.1.1. Classification Results

As observed in Table 3, the pre-trained models MobileNet, DenseNet201, and ResNet50V2 ranked top 3 on the prediction accuracy with 95.71%, 93.47%, and 93.47%, followed by ResNet101V2, ResNet152V2, MobileNetV2, and NASNet, all of which are achieved greater than 90% accuracy. Other models such as InceptionResNetV2, VGG16, Xception, InceptionV3, and VGG19 provided acceptable results with more than 80% accuracy. ResNet50 and ResNet101 achieved approximately 73% accuracy, whereas the worst outcomes were obtained by MobileNetV3Small and EfficientNetB7, which provided an accuracy of 50%. Other measures such as precision, recall, and F1 score are detailed in Table 3. 

#### 3.1.2. Confusion Matrix Results

**Figure 12:**
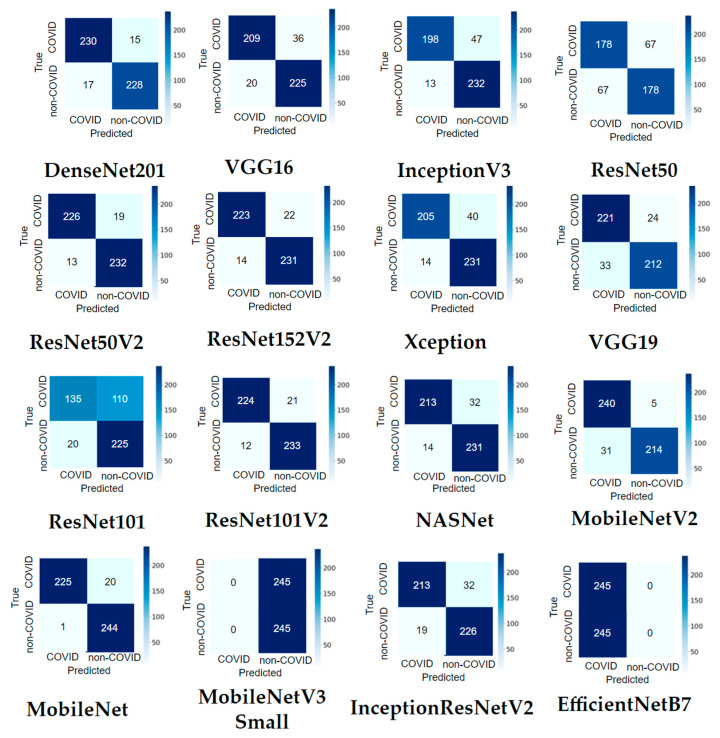
Confusion matrix result of the main dataset for sixteen modified pre-trained models in one hold-out run.

It can be observed from Figure 12 that most pre-trained models perform well in recognizing COVID-19 and non-COVID-19 CT scans. MobileNet only misclassified 21 out of 490 images (95.71% accuracy). ResNet50V2 and DenseNet201 made both 32/490 misclassifications (93.47% accuracy). MobileNet correctly identified 244 out of 245 non-COVID-19 images, and only one was misclassified (99.59% accuracy). MobileNetV2 recognized 240 of 245 COVID-19 images, with only five images that were not recognized (97.96% accuracy).

#### 3.1.3. Learning Curve Results

**Figure 13:**
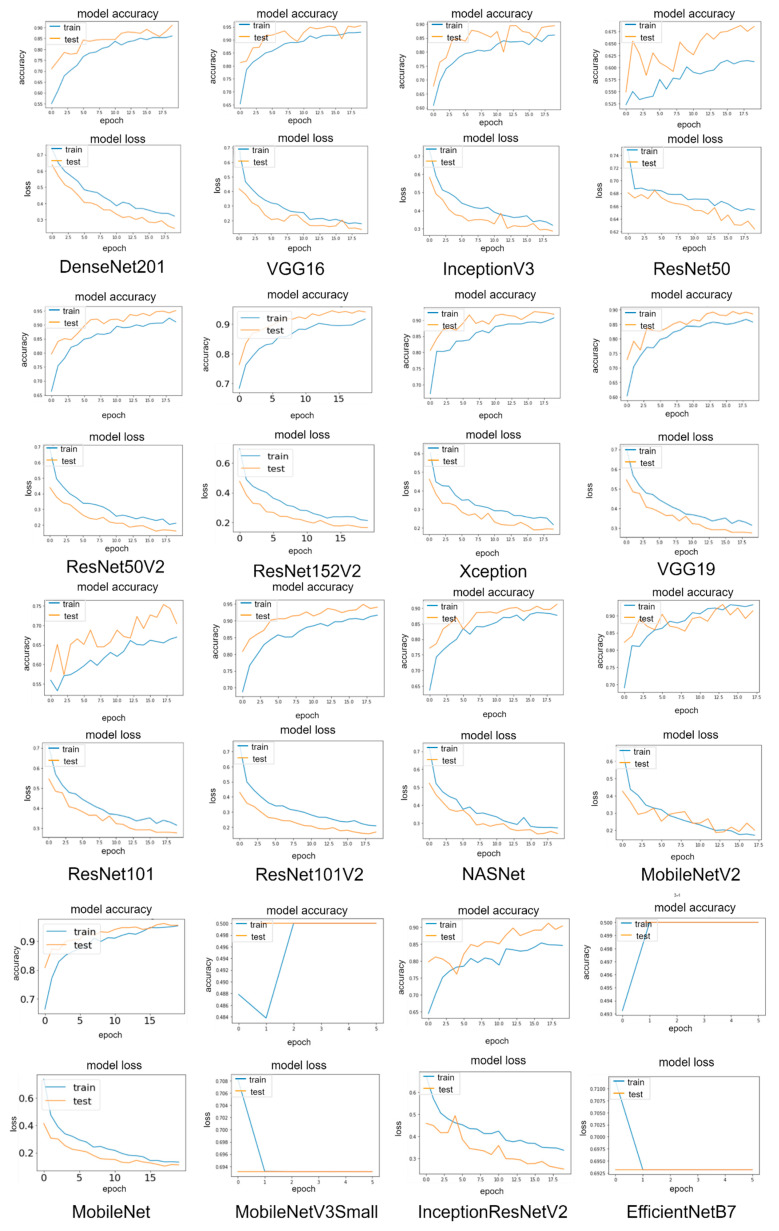
Learning curves of the main dataset for sixteen modified pre-trained models in one hold-out run.

Accuracy and loss curves for sixteen pre-trained models during training and validation periods are shown in Figure 13. The graphs show that the MobileNet has the lowest loss rate of 11.17% and the highest accuracy rate of 95.51%, followed by DenseNet201 and ResNet50V2 with loss rates of 15.93% and 22.59%, respectively. It can be observed that all validation curves exhibit oscillations compared to the training curve. This is because the size of the validation dataset is relatively small compared to the training dataset for the model to learn. The plot also indicated that all validation data resulted in better accuracy and a lower loss rate than the training data, suggesting that the models learn better on the validation dataset than the training dataset. This is because a dropout of 0.5 is used in model training, which means 50% of the features are set to zero, whereas all neurons are used in the validation, which results in better validation accuracy.

### 3.2. Results of EDNC Models

#### 3.2.1. Classification Results

Three ensemble strategies have been applied for recognizing COVID-19 CT images. It has been shown in Table 4 that all three EDNC models outperform individual pre-trained models in predicting COVID-19 lung infections. The accuracy, precision, specificity, and F1-score were improved by 4.08%, 6.03%, 5.72%, and 3.74%, respectively. However, pre-trained MobileNet still holds the most significant sensitivity of 99.56%. Among three ensemble models, the F-EDNC model obtained the best accuracy of 97.55%, and the highest recall of 96.41%. At the same time, FC-EDNC holds the highest F1-score of 97.18%, the highest specificity score of 98.33%, and the highest precision score of 98.37%. The CANet model received an accuracy of 91.63%, outperforming most of the pre-trained models. The classification results using the alternative dataset can be found in Table 5. It shows that the F-EDNC obtained the best accuracy of 97.83% and the highest sensitivity score of 100%. 

#### 3.2.2. Confusion Matrix Results

**Figure 14:**
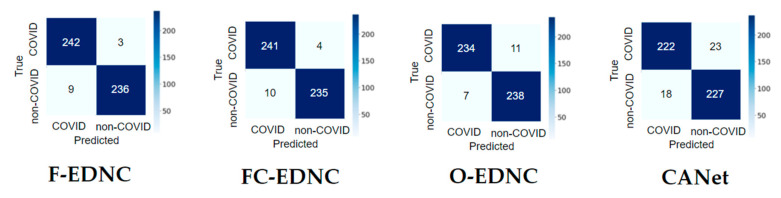
Confusion matrix result for EDNC and CANet models in one hold-out run.

It can be observed from the confusion matrix in Figure 14 that the numbers of misclassifications with proposed ensemble models have been significantly reduced compared to the numbers with the single pre-trained model. The proposed F-EDNC has only misclassified 12 CT scans out of 490 CT images (97.55% accuracy). The FC-EDNC model successfully classified 476 out of 490 CT images (97.14% accuracy), and O-EDNC correctly identified 472 CT images (96.32% accuracy). The CANet model misclassified 41 CT images (91.63% accuracy), which did not perform well compared to the ensemble models.

#### 3.2.3. False Discovery Rate Results

False discovery rate (FDR) means the percentage of all false discoveries, for example, the percentage of false discoveries in the calculation of all discoveries. The formula of FDR is as follows.

(19) $$\mathrm{FDR}=\frac{\mathrm{FP}}{\mathrm{TP}+\mathrm{FP}}$$

Table 6 indicates the FDR of three pre-trained models and all ensemble models, it can be observed that F-EDNC obtained the lowest FDR with 1.22%.

#### 3.2.4. Learning Curve Results

**Figure 15:**
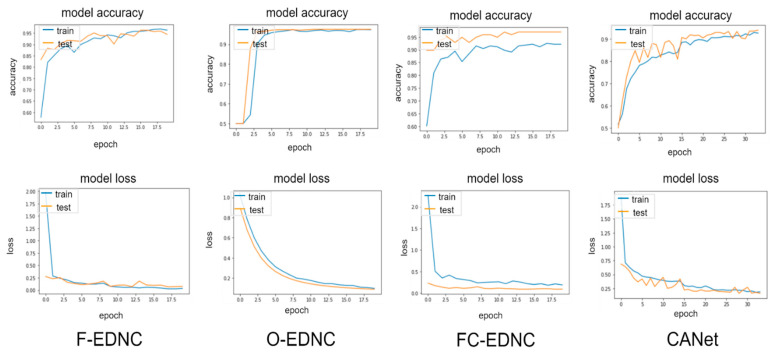
Learning curves for EDNC and CANet models in one hold-out run.

EDNC illustrates much better learning curves than individual pre-trained models, as shown in Figure 15. It can be observed that the F-EDNC model has the lowest loss rate of 3.42% and the highest accuracy rate of 98.92%, followed by O-EDNC and FC-EDNC with loss rates of 8.9% and 18.91% and accuracy rates of 96.89% and 92.18%, respectively. Furthermore, when training and validation loss decreases to a stable stage, the difference between the final training and validation values is minimal in F-EDNC and O-EDNC models, suggesting that F-EDNC and O-EDNC are good fit models. While in FC-ENDC, the gaps between the training and validation values in accuracy and lose curves are not promising.

The CANet model shows small gaps between validation and training value in the accuracy and loss curve; however, there are oscillations observed in the validation curve, which indicated that the size of the validation sample is too small for CANet to learn. According to the above results, it can conclude that F-EDNC performs best in categorizing chest CT scan images.

### 3.3. Classification Results of Five Runs for Pre-Trained Model and EDNC Model

To provide a more accurate assessment of model performance than a single validation (hold-out), we implement the process (random dataset-splitting, training, validation, and testing) five times on main dataset, with the findings averaged to obtain a more consistent and reliable result. Table 7 and Table 8 shows the averaged test results of proposed models in five hold-out runs. 

### 3.4. Training Time and Model Size Results

It can be observed from Table 9 that MobileNet used the smallest amount of time to complete one epoch training. At the same time, EfficientNetB7 consumed 34 s, which is the most considerable amount of time to finish one epoch training. The sizes of each model weight are shown in Table 4 as well. It can be observed that MobileNetV3Small has the smallest size of 13.26 MB, whereas the proposed F-EDNC has the most considerable model size of 377.2 MB.

### 3.5. Model Deployment Result

We deployed our COVID-19 recognition system in a localhost web application for users to use. The F-EDNC model was chosen to be deployed because it has the highest average of accuracy, precision, sensitivity, specificity, and F1-score.

**Figure 16:**
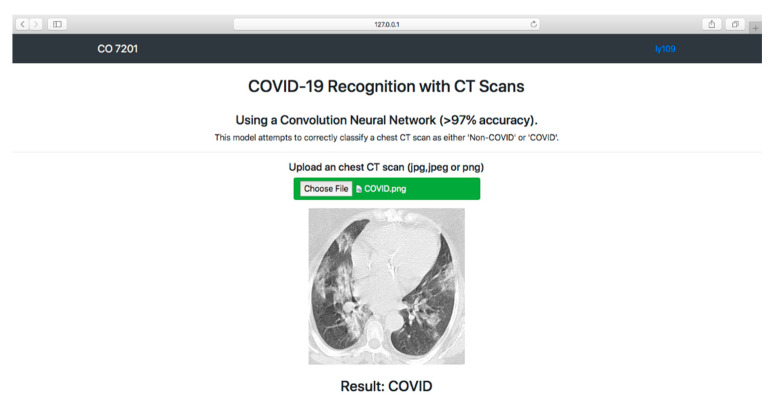
Overview of the frontend of the localhost web application.

As shown in Figure 16, a simple HTML web page was created to allow users to obtain COVID-19 results. Users can upload CT scan images by clicking the “Choose File” button. Once the users hit the “Predict!” button, the image will be sent to the system backend, where the proposed model can use the input image to predict the COVID-19 condition. The results (COVID-19 or Non-COVID-19) will be displayed on the front end. (The code is available at https://github.com/rgiol/code, access date: 4 January 2022).

## 4. Discussion

Using deep learning approaches for recognizing COVID-19 disease is a hot topic that has sparked much attention recently. In this field, exciting results have been shown and continue to emerge while simultaneously utilizing various neural networks. CT scan images are among the critical dataset types used to identify COVID-19 symptoms. Numerous deep learning models have been created and effectively deployed for identifying COVID-19.

In this study, twenty state-of-the-art techniques were selected for comparison purposes. After reviewing their methods and findings, we believe that there are still several research gaps in COVID-19 recognition compared to our study. Some of the most critical ones are listed as follows:

- The majority of studies utilized a dataset of only a few hundred COVID-19 images, which is inadequate for developing accurate and robust deep learning methods. Insufficient data may affect the performance of proposed methods.
- In most studies, there was a data imbalance problem, with one class having more images than the other. This affects the accuracy of models.
- Additionally, there are still some other pre-trained models that have not been utilized in COVID-19 classification.
- The impact of different ensemble methods has not received adequate attention in COVID-19 research. It should be emphasized that these techniques are beneficial in both improving performances and dealing with uncertainty associated with deep learning models.
- In none of the studies was there a webpage set up for users to upload images and to obtain COVID-19 predictions.

In contrast, our study used a perfectly balanced CT scan dataset with more than two thousand chest CT images. Sixteen pre-trained deep learning models have been investigated, including those not employed in the COVID-19 detection area. Furthermore, three ensemble models have been proposed to recognize COVID-19 CT images. The findings of each model are summarized in Table 10. The model proposed in our study outperforms most of the existing classifiers. 

## 5. Conclusions

This paper applies transfer learning methodology to modify and build sixteen deep learning models for COVID-19 recognition with the help of chest CT scans. Three ensemble deep neural networks (F-EDNC, FC-EDNC, and O-EDNC) were proposed further to enhance the performance of those sixteen deep learning models with a dataset containing 2458 CT scans. CANet, a self-build CNN model, has been designed and trained on the same dataset. The performances of the proposed EDNC have been evaluated and compared to CANet and the sixteen modified pre-trained models. The results have shown that EDNC outperformed the pre-trained models and CANet in COVID-19 image classification performance.

Among the results, F-EDNC achieves an accuracy of 97.75%, a sensitivity of 97.95%, a precision of 97.55%, a specificity of 97.56%, and an F1 score of 97.75%. Additionally, the proposed F-EDNC is deployed through a web application, enabling users to easily use the COVID-19 recognition system. Despite the excellent performance of the proposed COVID-19 recognition system, this study has several limitations. Firstly, if a user conducts the process of deriving a 2D image from a 3D CT scan, the classification result may vary depending on the selection of the 2D image. Secondly, this study has not utilized other preprocessing techniques such as image enhancement. In future work, image enhancement technology may be used to determine whether there is room for the improvement of results. In this study, the proposed EDNC significantly improved COVID-19 recognition performance, indicating the possibility of a completely automated and quick diagnosis of COVID-19 using deep learning. This finding will save time and money for health-care professionals in screening COVID-19 infections.

## Figures and Tables

**Table 1:** Information of the selected main Chest CT scan dataset.

Classes	Numbers of Samples	Format
COVID-19	1229	PNG
Non-COVID-19	1229	PNG

**Table 2:** Information of the selected alternative Chest CT scan dataset.

Classes	Numbers of Samples	Format
COVID-19	349	PNG
Non-COVID-19	349	PNG

**Table 3:** Result for modified pre-trained models of main dataset in one holdout run. Bold means these three models have the highest accuracy.

Model	Accuracy	Precision	Sensitivity	F1	Specificity
**DenseNet201**	**0.9347**	**0.9388**	**0.9312**	**0.9350**	**0.9383**
VGG16	0.8857	0.8531	0.9127	0.8819	0.8621
InceptionV3	0.8776	0.8082	0.9384	0.8684	0.8315
ResNet50	0.7265	0.7265	0.7265	0.7265	0.7265
**ResNet50V2**	**0.9347**	**0.9224**	**0.9456**	**0.9339**	**0.9243**
ResNet152V2	0.9265	0.9102	0.9409	0.9253	0.9170
Xception	0.8898	0.8367	0.9361	0.8836	0.8524
VGG19	0.8837	0.9020	0.8701	0.8858	0.8983
ResNet101	0.7306	0.5429	0.8693	0.6684	0.6716
ResNet101V2	0.9327	0.9143	0.9492	0.9314	0.9173
NASNet	0.9061	0.8694	0.9383	0.9025	0.8783
MobileNetV2	0.9265	0.9796	0.8856	0.9302	0.9772
**MobileNet**	**0.9571**	**0.9184**	**0.9956**	**0.9554**	**0.9242**
MobileNetV3Small	0.5000	0	0	0	0.5000
InceptionResNetV2	0.8959	0.8694	0.9181	0.8931	0.8760
EfficientNetB7	0.5000	1.0000	0.5000	0.6667	0

**Table 4:** Comparison of test result for pre-trained and ensemble model for main dataset in one holdout run. Bold indicates the model holds the highest accuracy.

Model	Accuracy	Precision	Sensitivity	F1	Specificity
ResNet50V2	0.9347	0.9224	0.9456	0.9339	0.9243
DenseNet201	0.9347	0.9388	0.9312	0.9350	0.9383
MobileNet	0.9571	0.9184	0.9956	0.9554	0.9242
**F-EDNC**	**0.9755**	**0.9787**	**0.9641**	**0.9713**	**0.9814**
O-EDNC	0.9632	0.9551	0.9710	0.9795	0.9630
FC-EDNC	0.9714	0.9837	0.9602	0.9718	0.9833
CANet	0.9163	0.9061	0.925	0.9155	0.908

**Table 5:** Comparison of test result for pre-trained and ensemble model for the alternative dataset in one holdout run. Bold means it outperformed other models.

Model	Accuracy	Precision	Sensitivity	F1	Specificity
ResNet50V2	0.9565	0.9710	0.9436	0.9571	0.9701
ResNet152V2	0.9510	0.9347	0.9347	0.9502	0.9673
MobileNet	0.9710	0.9420	1.0000	0.9701	0.9452
**F-EDNC**	**0.9783**	**0.9565**	**1.0000**	**0.9778**	**0.9583**
O-EDNC	0.9710	0.9420	1.0000	0.9701	0.9452
FC-EDNC	0.9783	0.9714	0.9602	0.9734	0.9602
CANet	0.9237	0.9168	0.9205	0.9223	0.9115

**Table 6:** False discovery rate (FDR) result for pre-trained and ensemble models for the main dataset. Bold shows it has the lowest FDR.

Model	FDR
ResNet50V2	0.0775
DenseNet201	0.0612
MobileNet	0.0816
**F-EDNC**	**0.0122**
O-EDNC	0.0448
FC-EDNC	0.0163
CANet	0.0938

**Table 7:** Average test result for pre-trained model for the main dataset in five runs. Bold means these three models have the highest accuracy.

Model	Accuracy	Precision	Sensitivity	F1	Specificity
**DenseNet201**	**0.9388**	**0.9610**	**0.9231**	**0.9412**	**0.9565**
VGG16	0.8918	0.8612	0.8792	0.8701	0.8692
InceptionV3	0.8734	0.8490	0.8489	0.8491	0.8560
ResNet50	0.7285	0.7224	0.7314	0.7269	0.7287
**ResNet50V2**	**0.9408**	**0.9306**	**0.9502**	**0.9403**	**0.9320**
ResNet152V2	0.9224	0.8980	0.9442	0.9205	0.9027
Xception	0.8939	0.8410	0.9406	0.8880	0.8571
VGG19	0.8776	0.8980	0.8627	0.8800	0.8936
ResNet101	0.7429	0.5673	0.8742	0.6881	0.6798
ResNet101V2	0.9306	0.9061	0.9527	0.9289	0.8764
NASNet	0.8980	0.8530	0.9372	0.8931	0.8652
MobileNetV2	0.9020	0.9836	0.8456	0.9094	0.9805
**MobileNet**	**0.9510**	**0.9383**	**0.9661**	**0.9520**	**0.9407**
MobileNetV3Small	0.5000	0	0	0	0.5000
InceptionResNetV2	0.9020	0.8776	0.9227	0.8996	0.8832
EfficientNetB7	0.5000	1.0000	0.5000	0.6667	0

**Table 8:** Average test result for pre-trained and ensemble model for the main dataset in five runs. Bold indicates the model holds the highest accuracy.

Model	Accuracy	Precision	Sensitivity	F1	Specificity
ResNet50V2	0.9408	0.9306	0.9502	0.9403	0.9320
DenseNet201	0.9388	0.9610	0.9231	0.9412	0.9565
MobileNet	0.9510	0.9383	0.9661	0.9520	0.9407
**F-EDNC**	**0.9775**	**0.9755**	**0.9795**	**0.9775**	**0.9756**
O-EDNC	0.9612	0.9592	0.9631	0.9611	0.9593
FC-EDNC	0.9755	0.9836	0.9679	0.9757	0.9834
CANet	0.9224	0.9347	0.9124	0.9234	0.9331

**Table 9:** Training time and weights comparison of all models in the main dataset.

Pre-Trained Model	Accuracy	Training Time (Second/Epoch)	Parameters (MB)
DenseNet201	0.9347	30	84.37
VGG16	0.8857	26	63.08
InceptionV3	0.8776	26	90.01
ResNet50	0.7265	27	103.99
ResNet50V2	0.9347	26	103.9
ResNet152V2	0.9265	28	237.5
Xception	0.8898	27	93.48
VGG19	0.8837	26	79.87
ResNet101	0.7306	28	177.19
ResNet101V2	0.9327	29	177.08
NASNet	0.9061	32	25.26
MobileNetV2	0.9265	27	17.52
MobileNet	0.9571	25	19.33
MobileNetV3Small	0.5000	28	13.26
InceptionResNetV2	0.8959	30	213.77
EfficientNetB7	0.5000	34	263.42
F-EDNC (Ours)	0.9755	31	377.2
O-EDNC (Ours)	0.9632	31	337.24
FC-EDNC (Ours)	0.9714	31	348
CANet (Ours)	0.9163	26	338.7

**Table 10:** Comparison with state-of-the-art approaches.

Author	Architecture	Accuracy	F1	Recall	Precision
Matsuyama, E. [17]	ResNet50 + wavelet coefficients	92.2%	91.5%	90.4%	/
Loey, M. [18]	ResNet50 + augumentation + CGAN	82.91%	/	77.66%	/
Do, C. [19]	Modified DenseNet201	85%	/	79%	91%
Polsinelli, M. [20]	Modified SqueezeNet	85.03%	86.20%	87.55%	85.01%
Panwar, H. [45]	Modified VGG19	94.04			
Mishra, A. [46]	Modified DenseNet121, ResNet50, VGG16, InceptionV3 and DenseNet201	88.3%	86.7%		90.15%
Ko, H. [21]	Modified VGG16, ResNet-50, Inception-v3, and Xception	96.97%			
Maghdid, H. [22]	Modified Alexnet, A self-build CNN	94.1%		100%	
Arora, V. [47]	Modified MobileNet, DenseNet121, ResNet50, VGG16, InceptionV3 and XceptionNet	94.12%	96.11%	96.11%	96.11%
Alshazly. H. [48]	CovidResNet and CovidDenseNet	93.87%	95.70	92.49	99.13%
Yu, Z. [49]	Modified InceptionV3, ResNet50, ResNet-101, DenseNet201	95.34%			
Jaiswal, A. [50]	Modified DenseNet201	96.25%	96.29%	96.29%	96.29%
Sanagavarapu, S. [51]	Ensembled ResNets	87%	84%	81%	91%
Song, J. [52]	A large-scale bi-directional generative adversarial network			92%	
Sarker, L [53]	Modified Densenet121	96.49%	96%	96%	96%
Shan, F. [54]	VB-Net	91.6%			
Wang, S. [55]	Modified DenseNet	85%	90%	79%	
Gozes, O. [56]	Modified ResNet50			94%	
Wang, S. [57]	Modified Inception	79.3%	63%	83%	
Li, L. [58]	Modified RestNet50			90%	
**Proposed**	**EDNC**	**97.75%**	**97.75%**	**97.95%**	**97.55%**

## Data Availability

https://www.kaggle.com/plameneduardo/sarscov2-ctscan-dataset (accessed on 20 August 2021).
